# Is Lamarckian evolution relevant to medicine?

**DOI:** 10.1186/1471-2350-11-73

**Published:** 2010-05-13

**Authors:** Adam E Handel, Sreeram V Ramagopalan

**Affiliations:** 1 Wellcome Trust Centre for Human Genetics, University of Oxford, Roosevelt Drive, Headington, Oxford, OX3 7BN, UK; 2 Department of Clinical Neurology, University of Oxford, The West Wing, John Radcliffe Hospital, Oxford, OX3 9DU, UK

## Abstract

**Background:**

200 years have now passed since Darwin was born and scientists around the world are celebrating this important anniversary of the birth of an evolutionary visionary. However, the theories of his colleague Lamarck are treated with considerably less acclaim. These theories centre on the tendency for complexity to increase in organisms over time and the direct transmission of phenotypic traits from parents to offspring.

**Discussion:**

Lamarckian concepts, long thought of no relevance to modern evolutionary theory, are enjoying a quiet resurgence with the increasing complexity of epigenetic theories of inheritance. There is evidence that epigenetic alterations, including DNA methylation and histone modifications, are transmitted transgenerationally, thus providing a potential mechanism for environmental influences to be passed from parents to offspring: Lamarckian evolution. Furthermore, evidence is accumulating that epigenetics plays an important role in many common medical conditions.

**Summary:**

Epigenetics allows the peaceful co-existence of Darwinian and Lamarckian evolution. Further efforts should be exerted on studying the mechanisms by which this occurs so that public health measures can be undertaken to reverse or prevent epigenetic changes important in disease susceptibility. Perhaps in 2059 we will be celebrating the anniversary of both Darwin *and *Lamarck.

## Background

The 200^th ^anniversary of the birth of Charles Robert Darwin FRS is rightly being celebrated across the world in 2009 [1,2]. Another event of interest in 1809, now largely forgotten, was the publication of Jean-Baptiste Pierre Antoine de Monet, Chevalier de la Marck's (Lamarck) *philosophie zoologique ou exposition des considérations relatives à l'histoire naturelle des animaux *[3]. Despite this work being the first formally published evolutionary theory, it garners little praise today. This was largely due to the widespread derision it received, most notably from Lamarck's colleague, Georges Cuvier [4]. Do Lamarck's ideas deserve to be forgotten? As nearly all current literature paints a disparaging view of Lamarck by referring to his supposition as to how giraffes acquired their characteristic morphology, it seems worthwhile to go back to review Lamarck's original texts. In this way we can assess the relevance of his thought on evolution to our current, ever expanding, knowledge of heredity though eyes unbiased by the modern, depreciatory view of his work.

*Philosophie zoologique *was studied by Stephen Jay Gould [4] among others. Lamarck's evolutionary theory can essentially be summarised with two concepts. The first is that organisms gradually and progressively become more complex by a "prime cause" and the second is that this progression is influenced by external conditions: "the environment exercises a great influence over the activities of animals, and as a result of this influence the increased and sustained use or disuse of any organ are causes of modification of the organization and shape of animals" [3,4]. This second concept leads to Lamarck's well known idea of acquired or soft inheritance: "the law of nature by which new individuals receive all that has been acquired in organization during the lifetime of their parents is so true, so striking...." [3,4].

## Discussion

After two centuries is there any evidence to support Lamarck's concepts? A number of studies do actually point to the existence of acquired characteristics and sometimes their inheritance. In rats, for example, stressed mothers have less time to care for offspring through postnatal licking/grooming (LG) and arched-back nursing (ABN) [5]. Low levels of postnatal LG and ABN result in offspring being more apprehensive, in order to cope with the stressful environment they have been born into (e.g. the presence of predators) [5]. In contrast, the offspring of high LG and ABN mothers are less fearful [5]. Cross-fostering between high and low LG and ABN mothers produces offspring with a fear phenotype as determined by their adoptive mother [5]. The phenotypes persist into adulthood, and females display the same behaviour as their mothers, thus propagating the trait [5]. Artificial, but nevertheless illustrative, is a study of gestating female rats exposed to endocrine disruptors [6]. These induce decreased spermatogenic capacity and increased incidence of infertility in male offspring. The phenotype is transferred through the male germ line all the way to males in the F4 generation [6].

How are characteristics 'acquired'? Epigenetics appears to provide the mechanism. A term coined by Waddington referring to changes in phenotype caused by any means other than changes in the underlying DNA sequence [7], epigenetics is now more directly interpreted as DNA and chromatin alterations that regulate genomic functions. These include DNA methylation of CpG dinucleotides and a multitude of different histone modifications [8]. Nearly all cells in the human body have the same genotype, but cells display hugely different phenotypes and this to some extent arises from epigenetics. The rules governing the establishment of epigenetic marks are not yet fully understood, but the epigenome is dynamic and the environment exerts a key influence over this. Epigenetic marks are therefore a reflection of an individual's environmental exposures and as such change during the lifetime of a cell/tissue [9]. Thus, we are 'acquiring' changes to our epigenome all the time.

Indeed, there is evidence that the Lamarckian experimental paradigms described above are directly mediated by epigenetic mechanisms. The more moderate stress responses of high LG and ABN rats is the result of an increase in the number of glucocorticoid receptors (GRs) in the hippocampus (which may be likened to Lamarck's use/disuse hypothesis as it is a consequence of increased serotonergic tone in the hippocampus) [5]. High LG and ABN rats have demonstrably lower levels of DNA methylation in the GR gene promoter region in hippocampal tissue resulting in increased GR expression, and this appears to be maintained for life [5]. Methylation of the GR promoter was a specific effect of exposure to maternal nurturing (i.e. environment) as the cross-fostering experiments showed that the effect on the epigenome was the same as for the true biological offspring [5].

For these epigenetic marks to behave as a Lamarkian concept, they must be transmitted between different generations of organisms. It had generally been thought that the epigenetic state of the genome is cleared between generations but there is increasing evidence that transgenerational epigenetic inheritance (and inheritance of acquired traits) occurs. In the study of endocrine disruptors, increased DNA methylation was observed at a number of gene regions in sperm from the F1 male rats as compared to controls, and this hypermethylation was inherited in the F2 and F3 generations [6]. It is likely that transgenerational transmission of epigenetic alterations is mediated by small molecules of RNA called microRNA that are capable of inducing sequence specific changes in the epigenome, although this has not yet been shown to modify the structure of chromatin [10].

Therefore, there is at least some evidence to support the various parts of Lamarck's concept of acquired inheritance. The field of epigenetics is very much in its infancy and therefore more examples are likely to follow. But is soft inheritance relevant to medicine? The answer is very likely to be yes. A number of key observations have recently come to light. Firstly, methylation levels vary between individuals and secondly, monozygotic twins are more similar than dizygotic twins in terms of levels of methylation [9,11]. Thus complex traits are likely a result of genetic, epigenetic and environmental components and their interactions. The classical genetic conundrums of incomplete penetrance and variable expressivity may in part be explained by differences in epigenetics. DNA methylation has already been highlighted as being important in aetiology for many common complex diseases, including cancer and psychosis [12-14]. Indeed, methylation of the GR promoter region is seen in suicide victims who were abused as children [15]. While this implicates acquired changes, there are also examples of inheritance, the most convincing being the transmission of a cancer associated epimutation in the *MLH *gene from a mother to one of three sons [16]. Epidemiological studies have also provided evidence highly suggestive of acquired epigenetic transgenerational aetiology. Looking at three generational families, Pembrey and co-workers showed that a paternal grandfather's food supply was linked to the mortality risk of his grandsons, while a paternal grandmother's food supply was linked to the mortality risk of her granddaughters [17]. Epigenetics provides probable mechanisms for these effects [18-20]. Thus, in humans as well as in experimental animal models, there is evidence that epigenetics provides a potential Lamarckian mechanism of evolution.

## Summary

As Dobzhansky observed "nothing in biology makes sense except in the context of evolution" [21]. It might be speculated that if nature could find a way to transmit ancestral environmental experience or stress to the benefit of the offspring she put it in play. While there may not be as yet many examples fitting Lamarck's theories perfectly, there is now enough evidence to concede that Lamarckian concepts have some merit. Epigenetics might actually be seen as unsurprising from an evolutionary aspect: one would expect intense selective pressure to be directed towards preserving any mechanism of maximising phenotypical variation and thus allows Darwinian evolution and Lamarckian theory (which Darwin accepted) [4] to co-exist peacefully. Indeed, a recent paper reporting an epigenetic model of evolutionary change has shown that methylation at CpG islands could optimise phenotypic variability and so lead to increased fitness [22]. Soft inheritance provides intriguing public health implications [23], as the fact that an individual's behaviours can affect the next two generations is rarely considered [24]. The implications to search for environmental factors especially in the ubiquitous case-control study is enormous. The most exciting part of epigenetics in modern medicine is the possibility of intervening at the junction between the genome and the environment, as unlike the DNA sequence, epigenetic changes are reversible. Research should now concentrate on revealing which conditions arise as a result of epigenetic changes and whether it is possible to intervene in this process to prevent disease or restore health. Perhaps in 2059, we will be celebrating 250 years of Darwin *and *Lamarck.

## Abbreviations

LG: licking and grooming maternal behavior in rats; ABN: arched back nursing maternal behavior in rats; F1, F2, F3, F4: number of generations after original gestating female; GR: glucocorticoid receptor; CpG islands: directly linked cytosine and guanosine residues

## Competing interests

The authors declare that they have no competing interests.

## Authors' contributions

AEH & SVR conceived the idea and wrote the manuscript. Both authors approved and read the manuscript.

## Pre-publication history

The pre-publication history for this paper can be accessed here:

http://www.biomedcentral.com/1471-2350/11/73/prepub
